# Round the Hospitals

**Published:** 1920-12-18

**Authors:** 


### ROUND THE HOSPITALS.

This week the attention of College members is called to the fact that the College is circulating the letter to Founder Members (*i.e.*, nurses registered before November 20, 1920) on the question of an annual subscription of 5s. decided upon at the recent general meetings. Accompanying it is also a *questionnaire* arising from General Nursing Council's reported vote against the nursing profession's inclusion in the Hours of Employment Bill under the Ministry of Labour, and their resolution that "the Minister of Health be asked to introduce a Bill to regulate the hours of nurses employed in hospitals and other institutions for care of the sick." This resolution would rule out the suggested Special Order supported by members of the College in connection with the Hours of Employment Bill, and a number of questions are therefore submitted for the members' consideration, together with the urgent request that they should be answered and returned to the Secretary without delay. The problem of inclusion of the nursing profession in the Unemployment Insurance Act is also dealt with.

As a result of a report to the Ministry of Health by Dr. Manby, one of their medical officers, the Southwark Board of Guardians have decided to make a series of alterations and improvements at their maternity wards in the Newington Institution. Unfortunately, these alterations were not decided upon until the attention of the guardians was drawn to the death of a seventeen-year old girl in the wards. After a full investigation the guardians came to the conclusion that Dr. J. F. Williams, the medical officer at the institution, to the best of his judgment and skill, did all he possibly could to save the life of this patient, and they expressed their utmost confidence in him, based upon thirty-seven years' experience in charge of the institution. The guardians, however, decided that Dr. Williams should be authorised to call in a consultant specialist at any time he considered it desirable: that in any future appointments of midwives only applications from nurses with a certificate of three years' training in a recognised training-school for nurses, and holding the certificate of the Central Midwives Board, he considered, and that the existing staff of midwives be replaced by nurses with these qualifications as early as possible. The Visiting Committee were instructed to consider the provision of trained nurses for any serious case isolated or transferred from the maternity wards.

Lady Waley Cohen is kindly lending her residence "Caen Wood Towers," Hampstead Lane, for a Children's Fancy Dress Dance on Saturday, January 8 next, from 3.30 to 9.30 p.m. Dancing displays will be given by professional dancers and the pupils of Miss Shotter. The dance is under the auspices of the Ladies' Association of the Great Northern Hospital, and the proceeds are in aid of the general funds of that institution.

Princess Louise, Duchess of Argyll, opened the two days' fair organised by the Kensington Surgical Supply Depot at 29 Kensington Court, and a very successful sale ensued. New premises are an urgent need if this admirable organisation is to continue its work, and these will cost £6,500. One of the specialities of the depot is the manufacture of artificial limbs and surgical appliances, and great interest was displayed in specimens of these articles on view. During the war the value of the stuff turned out at the depot amounted to £350,000. To continue these labours for "the victims of peace" is the aim of these voluntary workers, and the sooner they get into permanent quarters the better it will be for the hospitals.

In his annual report Dr. Edmund M. Smith, York city medical officer of health, mentions that during recent years they have made a very determined effort in the prevention of ophthalmia neonatorum, which is mostly due to gonorrhœa. Over and over again the certified midwives have been circularised and addressed upon the subject, and as there are old-fashioned ideas associating the cause with "chill," they have had to make persistent efforts to eliminate from the minds of the older women such mistaken notions. For several years past they have been supplying Protargol drops gratis to midwives, for preventive use soon after birth.

"The debt this nation owes to the Queen's nurses," said Lady Cory, "is never fully realised and can never be repaid." The statement was made at a social gathering at Cardiff last week in aid of the Needlework Guild of the Cardiff Queen's nurses, when much was spoken in praise of these splendid women and the splendid work they do. Canon Davies added his tribute to the value of the nurse in the community, and remarked that he made it a habit when a curate in the early days of his ministry to cultivate the acquaintance of as many nurses as possible. There are a dozen and a-half nurses attached to the Cardiff Queen's Nurses' Institute, and last year they attended over 1,800 cases.

At a monthly meeting of the Royal Portsmouth Hospital the following increases in salary were granted: Assistant matron, £70, rising by yearly increment of £5 to £90; sister-tutor, £80, rising to £90; *x*-ray sister, £70, rising to £80; theatre and casualty sisters, £60, rising to £80; night sister, £70; ward sisters, £60, rising to £80.

The Royal Victoria Hospital, Belfast, is faced with an alarming deficit, the debt having nearly trebled and expenses continuing to mount. Part of the additional expenditure is attributed to the nursing department, where there are forty-four more nurses on the staff than in 1913. New departments have been opened, and the activities of the hospital largely increased, so it need not be imagined that the nursing staff has nothing to do now as compared with pre-war times.

